# Supplementary material for: Intercellular adhesion molecule-1 protects against adipose tissue inflammation and insulin resistance but promotes liver disease activity in western-diet fed mice
Source: Sci Rep. 2025 Jul 17;15:25884. doi: 10.1038/s41598-025-11555-0 (PMC12267403; doi:10.1038/s41598-025-11555-0)
Supplement: Supplementary file 1 — Supplementary Material 1 [file 41598_2025_11555_MOESM1_ESM.docx]

Supplementary Material

**Intercellular adhesion molecule-1 protects against adipose tissue inflammation and insulin resistance but promotes liver disease activity in western-diet fed mice**

Sreepradha Eswaran, Laura Gebert, Sarah Schraven, Nicole Treichel, Thomas Ritz, Sabine Hamm, Agnes Seeger, Fabian Kiessling, Thomas Clavel, Stephan Dreschers, Norbert Wagner, Angela Schippers

**Supplementary Figures:**

**Supplementary Figure S1:**

**
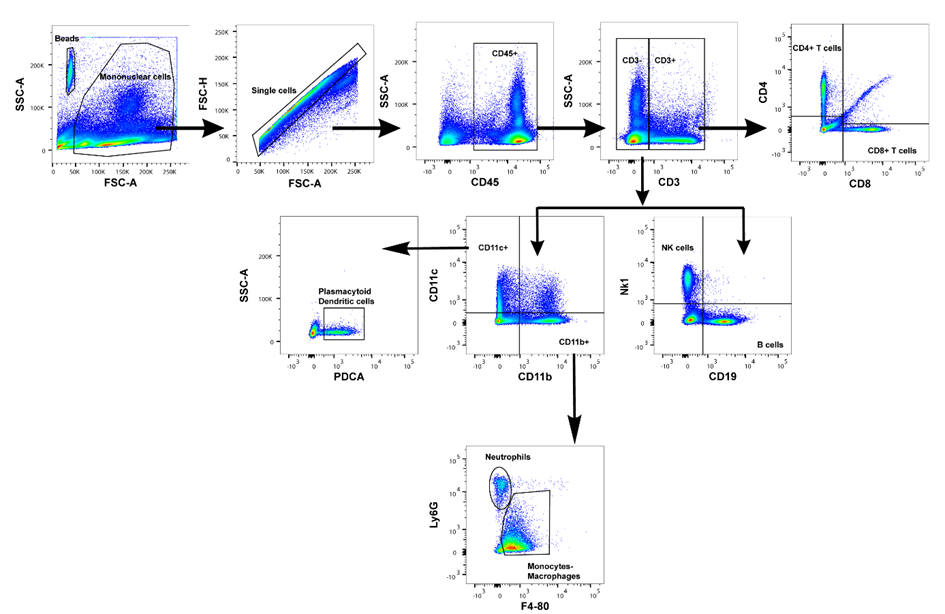
**

**Supplementary Figure S1.** Immune cell subset gating by multiparameter flow cytometry (liver cells, representative of all organ fractions). The analysis included: B cells (CD45^+^CD3^‐^CD19^+^), CD4^+^ T cells (CD45^+^CD3^+^CD4^+^), CD8^+^ T cells (CD45^+^CD3^+^CD8^+^), natural killer (NK) cells (CD45^+^ CD3^‐^NK1.1^+^), monocytes/macrophages (Mo‐MF)(CD45^+^CD11b^+^Ly6G^‐^ F4/80^+^), neutrophils (CD45^+^CD11b^+^ CD11c^-^F4/80^-^Ly6G^+^), and plasmacytoid dendritic cells (pDCs) (CD45^+^ CD11b^+^CD11c^+^PDCA^+^).

**Supplementary Figure S2:**

**
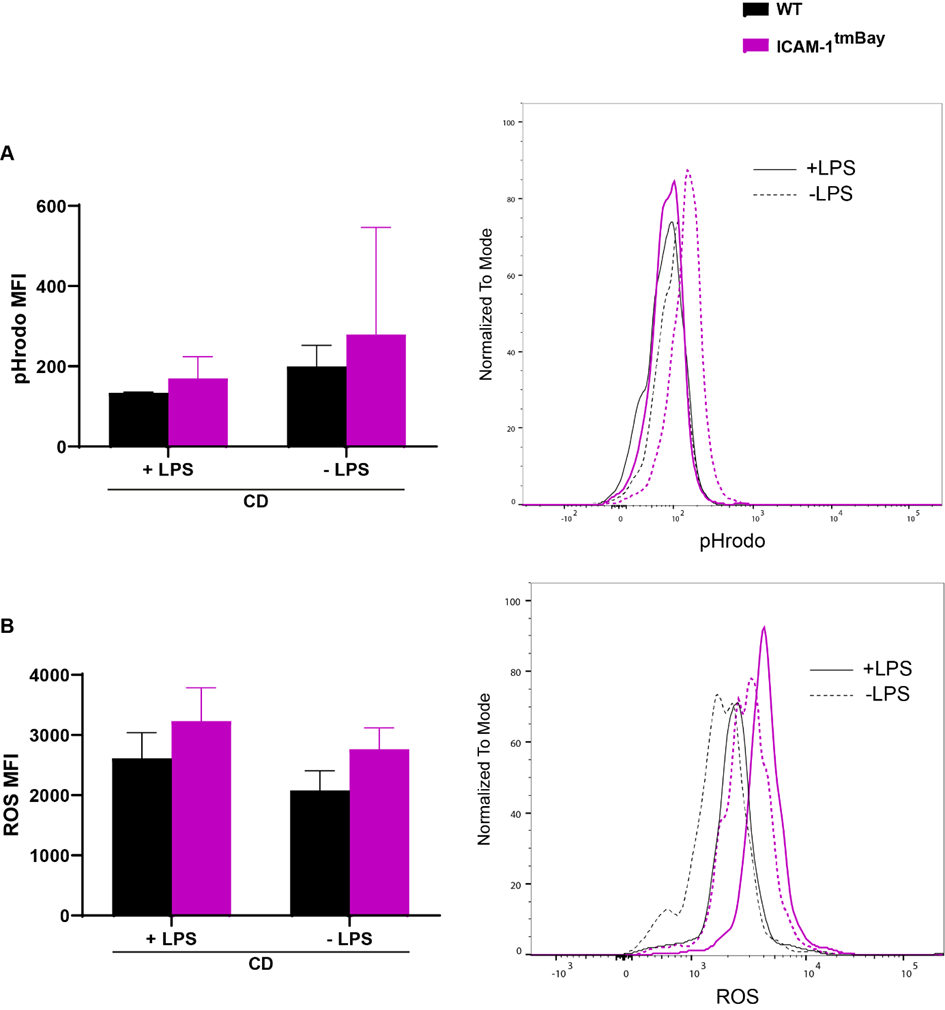
**

**Supplementary Figure S2.** Flow cytometric analysis of pHrodo and reactive oxygen species (ROS) in LPS-stimulated or unstimulated CD45^+^CD11b^+^Gr1^+^ myeloid cells of whole blood from chow-fed (CD) WT mice (shown in black, n = 3) and ICAM-1 mutant (Icam1^tmBay^) mice (shown in purple, n = 5). (A) Mean fluorescence intensity (MFI) of pHrodo and representative histograms. (B) Mean fluorescence intensity (MFI) of ROS and representative histograms. Statistical significance was calculated by two-way ANOVA (A-B). Values are represented as mean ± SD.

**Supplementary Figure S3:**


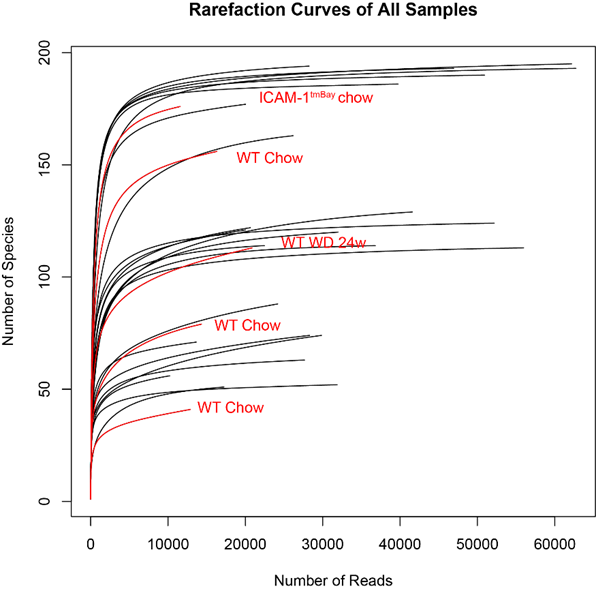


**Supplementary Figure S3.** Rarefaction curves depicting sequencing depth. The number of observed molecular species in each sample was plotted against the number of reads acquired, to ensure sufficient sequencing depth. The curve was generated in R using Rhea ^1^. The five samples with the least sequencing reads are shown in red.

**Supplementary Figure S4:**

**
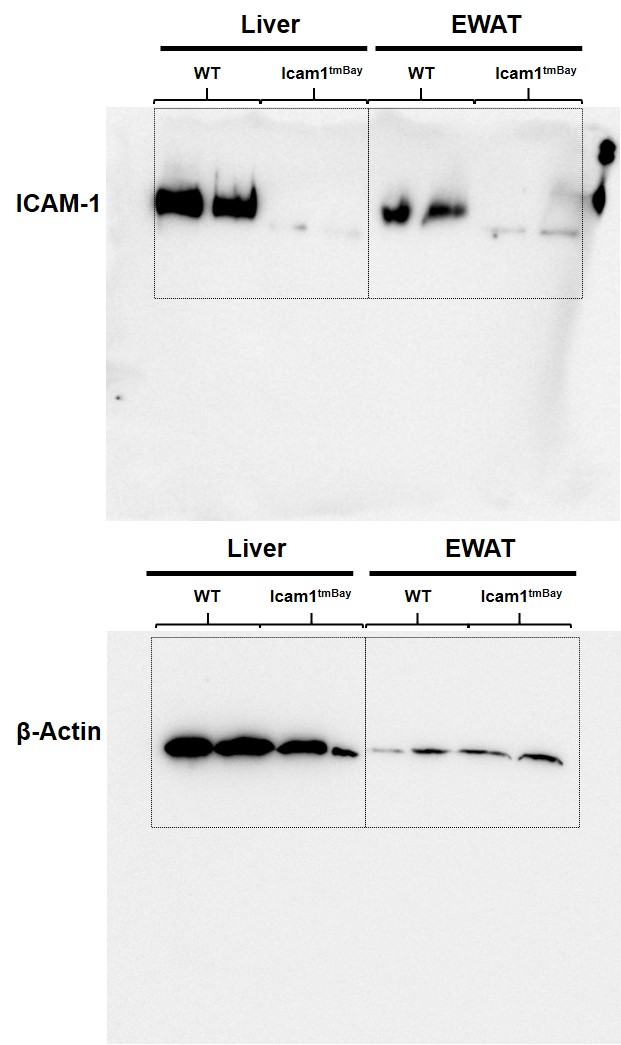
**

**Supplementary Figure S4.** Western blot to check for the ICAM-1 mutation. Protein extracts from liver and epidydimal white adipose tissue (EWAT) of two WT and two Icam1^tmBay^ animals were analyzed for the presence of ICAM-1 protein. β-actin was used as a loading control.

**Supplementary Tables:**

**Supplementary Table S1: Ingredients of the Western-style diet**

| **Class description** | **Ingredients** | **Grams** |
| --- | --- | --- |
| **Protein** | Casein, Lactic, 30 Mesh | 200.00 g |
| **Protein** | Cystine, L | 3.00 g |
| **Carbohydrate** | Fructose | 200.00 g |
| **Carbohydrate** | Sucrose, Fine Granulated | 100.00 g |
| **Carbohydrate** | Lodex 10 | 100.00 g |
| **Fiber** | Solka Floc, FCC200 | 50.00 g |
| **Fat** | Primex Shortening-Z (non-transfat) | 135.00 g |
| **Fat** | Soybean Oil, USP | 25.00 g |
| **Fat** | Lard | 20.00 g |
| **Mineral** | S10026B | 50.00 g |
| **Vitamin** | Choline Bitartrate | 2.00 g |
| **Vitamin** | V10001C | 1.00 g |
| **Special** | Cholesterol, NF | 18.00 g |
| **Dye** | Dye, Red FD&C #40, Alum. Lake 35-42% | 0.05 g |
|  | Total: | 904.05 g |

**Reference :** [**https://researchdiets.com/en/formulas/d16022301**](https://researchdiets.com/en/formulas/d16022301)

**Supplementary Table S2:** Antibodies for histochemical analysis

| Antibody | Channel/Conjugate | Manufacturer | Order No | Dilution |
| --- | --- | --- | --- | --- |
| ICAM-1 | Pure | Invitrogen | MA5-43105 | 1:50 |
| MPO | Pure | Bioss | bs4943R | 1:100 |
| CD68 | Pure | Abcam | ab125212 | 1:1000 |
| Goat anti-rabbit IgG | Biotin | VectorLabs | BA-1000 | 1:200 |

ICAM-1, Intercellular adhesion molecule 1; MPO, Myeloperoxidase.

**Supplementary Table S3:** Antibodies used for flow cytometric analysis

| Antibody | Channel/Conjugate | Manufacturer | Clone | Dilution |
| --- | --- | --- | --- | --- |
| NK 1.1 | AF 488 | Biolegend | PK136 | 1:400 |
| CD4 | PE | BD biosciences | GK1.5 | 1:400 |
| CD45 | APC-Cy7 | BD biosciences | 30-F11 | 1:400 |
| CD19 | efluor 450 | eBioscience | 1D3 | 1:200 |
| CD8a | Amcyan | Biolegend | 53-6.7 | 1:400 |
| CD3e | PerCp-Cy5.5 | eBioscience | 145-2C11 | 1:100 |
| F4/80 | efluor450 | Serotec | CI:A3-1 | 1:200 |
| CD11b | Amcyan | BD Biosciences | M1/70 | 1:400 |
| Ly6G | FITC | BD Biosciences | 1A8 | 1:400 |
| CD317 | PE | eBioscience | eBio129c | 1:400 |

AF 488, Alexa Fluor 488; PE Phycoerythrin; APC-Cy7, Allophycocyanin-Cyanine 7; FITC Fluorescein isothiocyanate.

**Supplementary Table S4:** Primers used in this study

| Primer | Forward | Reverse |
| --- | --- | --- |
| *Gapdh* | acctgccaagtatgatgacatca | ggtcctcagtgtagcccaagat |
| *Icam-1* | cacccaccccgcaggtcca | ttccccaagcagtccgtctcg |
| *Mcp-1* | agagccagacgggaggaag | ccagcctactcattgggatc |
| *Col1a* | gcagggttccaacgatgttg | gcagccatcgactaggacaga |

*Gapdh*, Glyceraldehyde 3-phosphate dehydrogenase; *Icam-1*, Intercellular adhesion molecule 1; *Mcp-1*, Monocyte chemoattractant protein-1; *Coll1a*, Collagen type I alpha.

**Supplementary References**

1 Lagkouvardos, I., Fischer, S., Kumar, N. & Clavel, T. Rhea: a transparent and modular R pipeline for microbial profiling based on 16S rRNA gene amplicons. *PeerJ* **5**, e2836 (2017). https://doi.org/10.7717/peerj.2836
